# Cefmetazole-Induced Vitamin K–Dependent Coagulopathy Associated with Life-Threatening Gastrointestinal Bleeding in a Critically Ill Older Patient: A Case Report

**DOI:** 10.70352/scrj.cr.26-0417

**Published:** 2026-07-14

**Authors:** Takamichi Nishida, Daisuke Muroya, Hisaaki Shimokobe, Satoshi Taniwaki

**Affiliations:** 1 Department of Surgery, Tobata Kyoritsu Hospital, Kitakyushu, Fukuoka, Japan; 2 Department of Emergency Medicine, Kitakyushu City Yahata Hospital, Kitakyushu, Fukuoka, Japan

**Keywords:** cefmetazole, vitamin K–dependent coagulopathy, gastrointestinal bleeding, critically ill older patient, intensive care unit (ICU)

## Abstract

**INTRODUCTION:**

Cefmetazole (CMZ) is frequently used in abdominal surgery and critical care settings. Although cephalosporins containing an N-methyl-tetrazole-thiol (NMTT) side chain interfere with vitamin K metabolism, life-threatening clinically significant bleeding associated with CMZ-induced coagulopathy has rarely been reported.

**CASE PRESENTATION:**

A 78-year-old woman with severe infections developed severe vitamin K–dependent coagulopathy during CMZ therapy. She subsequently experienced worsening upper gastrointestinal bleeding complicated by hemorrhagic shock in the setting of severe vitamin K–dependent coagulopathy despite preserved platelet counts and fibrinogen levels. Coagulation abnormalities rapidly improved following vitamin K administration, and no recurrence was observed after CMZ discontinuation.

**CONCLUSIONS:**

This case report highlights CMZ-induced vitamin K–dependent coagulopathy as a rare but life-threatening adverse event in critically ill older patients. Coagulation parameters should be carefully monitored during CMZ therapy, particularly in high-risk patients.

## Abbreviations

APTT
: activated partial thromboplastin time
CHDF
: continuous hemodiafiltration
CMZ
: cefmetazole
CPZ
: cefoperazone
CTRX
: ceftriaxone
DIC
: disseminated intravascular coagulation
FFP
: fresh frozen plasma
HD
: hospital day
IV
: intravenous
JAAM
: Japanese Association for Acute Medicine
MEPM
: meropenem
NE
: norepinephrine
NMTT
: N-methyl-tetrazole-thiol
PIVKA-II
: protein induced by vitamin K absence or antagonist-II
PPI
: proton pump inhibitor
PT-INR
: prothrombin time–international normalized ratio
RBC
: red blood cells

## INTRODUCTION

CMZ is frequently used in abdominal surgery and critical care settings. Cephalosporins containing an NMTT side chain that interferes with vitamin K metabolism may cause coagulation disorders. However, life-threatening bleeding events associated with CMZ have rarely been reported. Here, we describe the case of a critically ill older patient who developed severe vitamin K–dependent coagulopathy during CMZ therapy, with subsequent worsening of pre-existing gastrointestinal bleeding and hemorrhagic shock.

## CASE PRESENTATION

A 78-year-old woman with a history of pelvic fracture and no antithrombotic medication use presented to the orthopedic outpatient clinic at Tobata Kyoritsu Hospital with atraumatic right hip pain. MRI revealed a right pubic insufficiency fracture, and the patient was discharged. Three days later, she developed loss of appetite and progressive difficulty with ambulation. Four days after the initial visit, she was transferred to the emergency department because of severe spontaneous pain and marked tenderness extending from the right pubic region to the medial aspect of the right thigh.

CT revealed right pubic osteomyelitis complicated by a gas-forming abscess involving the right adductor muscle. Laboratory findings revealed systemic inflammation and organ dysfunction, and the patient was diagnosed with sepsis complicated by acute kidney injury and sepsis-associated coagulopathy (**Fig. 1** and **Table 1**). Empiric antimicrobial therapy with MEPM was initiated, and emergency incision and drainage of the gas-forming adductor muscle abscess were performed.

**Fig. 1:**
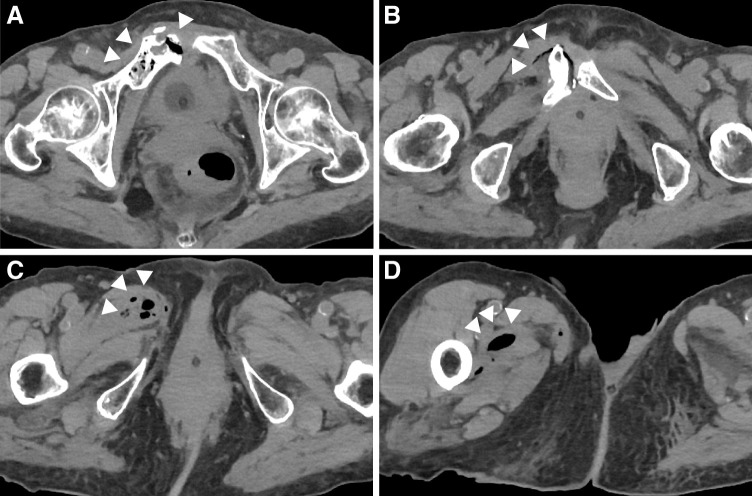
Non-contrast CT findings on admission. (**A**) Gas accumulation is observed within the right pubic bone and around the previous fracture site (white arrowheads). (**B**) An abscess with surrounding gas formation is observed around the pubic symphysis (white arrowheads). (**C**, **D**) Extensive intramuscular gas accumulation is observed in the right adductor muscle group (white arrowheads).

**Table 1:** Laboratory findings on admission

Category	Parameter	Value	Reference range
Inflammation	White blood cell count (/μL)	19850	3300–8600
	C-reactive protein (mg/dL)	21.67	0.00–0.14
Renal function	Blood urea nitrogen (mg/dL)	136.3	8.0–20.0
	Creatinine (mg/dL)	4.52	0.46–0.79
Liver function	Total bilirubin (mg/dL)	0.52	0.40–1.50
	Aspartate aminotransferase (U/L)	29	13–30
	Alanine aminotransferase (U/L)	25	7–23
	Alkaline phosphatase (U/L)	148	38–113
Hematology	Hemoglobin (g/dL)	12.4	11.6–14.8
	Platelet count (×10^3^/μL)	55	158–348
Coagulation	PT-INR	1.08	0.8–1.0
	APTT (s)	28.4	15.0–45.0
	D-dimer (μg/mL)	18.0	<1.0
Nutrition	Total protein (g/dL)	5.3	6.6–8.1
	Albumin (g/dL)	2.2	4.1–5.1

APTT, activated partial thromboplastin time; PT-INR, prothrombin time–international normalized ratio

After the initial surgery, the patient was admitted to the ICU and required continuous NE infusion for septic shock. Oral intake was resumed on HD 2, and a PPI was initiated for stress ulcer prophylaxis. CHDF was initiated on HD 3 because of persistent acute kidney injury, and septic shock subsequently resolved.

Upper gastrointestinal bleeding developed on HD 6. Esophagogastroduodenoscopy revealed multiple gastric and duodenal ulcers without evidence of active bleeding, and 2 units of RBCs were transfused (**Fig. 2**). PPI therapy was switched to IV administration. On HD 7, radiography and CT revealed an upper gastrointestinal perforation. As the abdominal pain was localized to the right upper quadrant and the patient remained hemodynamically stable, nonoperative management was initially selected.

**Fig. 2:**
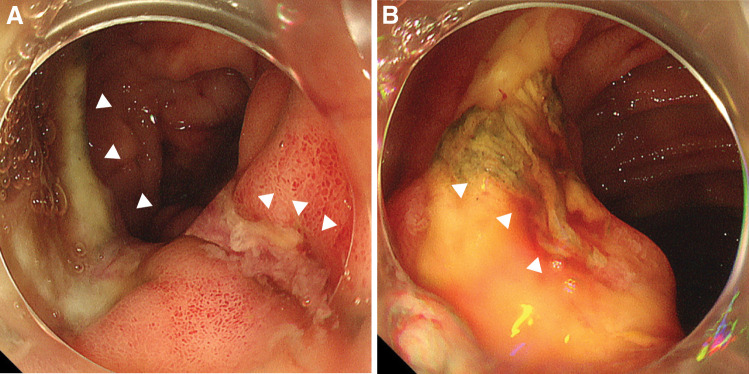
Emergency esophagogastroduodenoscopy findings on hospital day 6. (**A**) Stomach and (**B**) duodenum. Multiple gastric and duodenal ulcers (white arrowheads) were observed without evidence of active bleeding.

On HD 9, cultures obtained from urine, blood, and intraoperative abscess specimens yielded *Escherichia coli* with favorable antimicrobial susceptibility profiles, and antimicrobial therapy was de-escalated to CMZ. On HD 10, worsening abdominal pain prompted laparoscopic surgery, during which a simple closure and omental patch repair of the duodenal bulb perforation were performed (**Fig. 3**). CMZ was temporarily discontinued on HD 15. However, because prolonged antimicrobial therapy was deemed necessary for right pubic osteomyelitis, CMZ was reinitiated on HD 16.

**Fig. 3:**
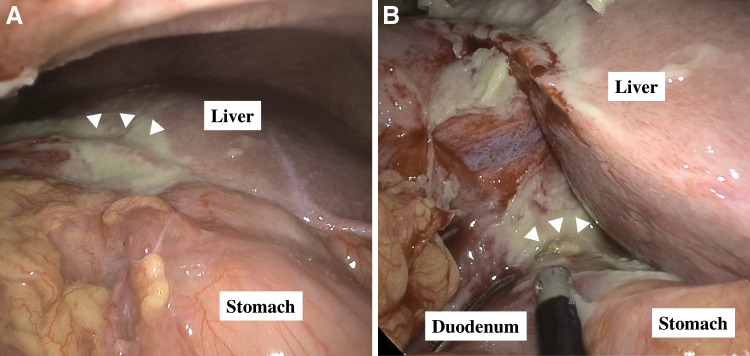
Intraoperative findings on hospital day 10. (**A**) Laparoscopic view showing turbid ascites in the right subphrenic space with adhesions between the liver and surrounding tissues. Prominent fibrinous exudates were also observed (white arrowheads). (**B**) A perforation measuring approximately 5 mm in diameter was identified on the anterior wall of the duodenal bulb (white arrowheads).

On HD 22, the patient developed hemorrhagic shock secondary to life-threatening upper gastrointestinal bleeding. Laboratory testing revealed marked prolongation of coagulation parameters, with a PT-INR of 12, indicating severe coagulopathy (platelet count: 378 × 10^3^/µL; fibrinogen: 299 mg/dL; D-dimer: 4.4 µg/mL). The patient received transfusions of FFP and RBCs along with empiric IV vitamin K (20 mg). Emergency endoscopy revealed active bleeding from an exposed vessel in the duodenal bulb, which was successfully treated endoscopically (**Fig. 4**). CMZ-induced vitamin K–dependent coagulopathy was strongly suspected, and antimicrobial therapy was switched to CTRX. Subsequent laboratory analysis, which became available after the acute bleeding episode, demonstrated a markedly elevated PIVKA-II level of 51024 mAU/mL, further supporting the diagnosis. Follow-up endoscopy confirmed sustained hemostasis, healing of all ulcers, and closure of the previous perforation site on HD 23. Oral intake was resumed on HD 24, and no recurrence of gastrointestinal bleeding or coagulopathy was observed. The patient was transferred to a rehabilitation facility on HD 72 without recurrence of gastrointestinal bleeding or coagulopathy (**Fig. 5**).

**Fig. 4:**
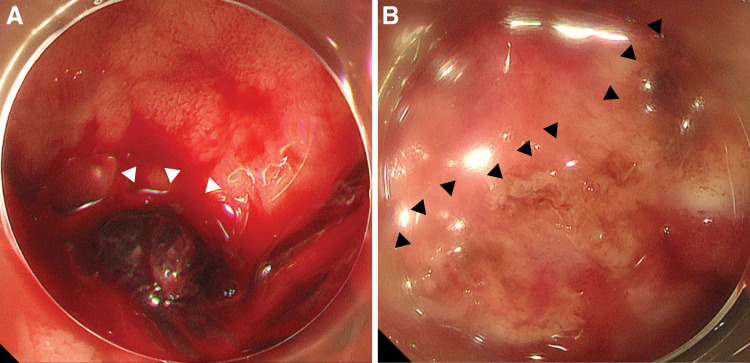
Emergency esophagogastroduodenoscopy findings on hospital day 22. (**A**) Active bleeding from an exposed vessel in a duodenal ulcer (white arrowheads). (**B**) Oozing hemorrhage from multiple gastric and duodenal ulcers (black arrowheads), treated with endoscopic hemostatic procedures as appropriate.

**Fig. 5:**
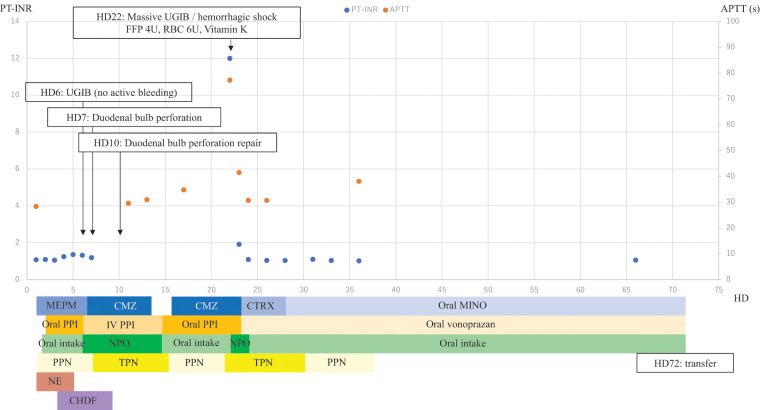
Clinical timeline of coagulation parameters and key interventions. Changes in PT-INR (left y-axis) and APTT (right y-axis) during hospitalization are shown. The x-axis indicates HDs. Antimicrobial therapy, acid-suppressive therapy, nutritional support, organ support, and major clinical events are shown. APTT, activated partial thromboplastin time; CHDF, continuous hemodiafiltration; CMZ, cefmetazole; CTRX, ceftriaxone; FFP, fresh frozen plasma; HD, hospital day; IV, intravenous; MEPM, meropenem; MINO, minocycline; NE, norepinephrine; NPO, *nil per os*; PPI, proton pump inhibitor; PPN, peripheral parenteral nutrition; PT-INR, prothrombin time–international normalized ratio; RBC, red blood cells; TPN, total parenteral nutrition; UGIB, upper gastrointestinal bleeding

## DISCUSSION

This case highlights a rare but serious complication of CMZ therapy in a critically ill older patient with severe infections, in whom vitamin K–dependent coagulopathy developed and was associated with life-threatening gastrointestinal bleeding. CMZ is a cephamycin antimicrobial containing an NMTT side chain, a structural feature shared with other cephalosporins such as CPZ. Cephalosporins containing an NMTT side chain are known to inhibit vitamin K epoxide reductase, thereby impairing the recycling of active vitamin K and prolonging PT and PT-INR.^1)^ In addition, the disruption of the intestinal microbiota associated with antimicrobial therapy reduces endogenous vitamin K production. Prolonged fasting, malnutrition, and critical illness may further reduce dietary vitamin K intake. Importantly, the reported incidence of CMZ-induced coagulopathy is 5.7% among patients treated with CMZ for intra-abdominal infections, indicating that this adverse effect is relatively common in clinical practice.^2)^

In the present case, the patient was an older adult and severely infected, with poor nutritional status at admission. She required intensive care management, including CHDF, broad-spectrum antimicrobial therapy, and prolonged fasting due to gastrointestinal bleeding and perforation. These predisposing factors likely acted synergistically to precipitate severe vitamin K–dependent coagulopathy during CMZ therapy. DIC was also considered in the differential diagnosis; however, this was deemed unlikely because the JAAM-2 DIC diagnostic criteria yielded a total score of 1. Furthermore, the prompt improvement in coagulopathy following vitamin K administration, together with the absence of recurrence after discontinuation of CMZ, strongly suggested that CMZ-induced vitamin K–dependent coagulopathy aggravated the bleeding tendency and contributed to the development of hemorrhagic shock.

A review of previously reported cases suggests that CMZ-induced coagulopathy predominantly occurs in older patients and often manifests as gastrointestinal bleeding.^2)^ Previous reports diagnosed vitamin K–dependent coagulopathy based on prolonged PT/APTT, mixing studies, and clinical responses to vitamin K supplementation.^3)^ In contrast, several reports from Japan demonstrated vitamin K–dependent coagulopathy by measuring PIVKA-II. In the present case, the PIVKA-II level was markedly elevated to 51024 mAU/mL during the life-threatening gastrointestinal bleeding, consistent with previous findings.^1,2)^

Previous reports have shown that CMZ-induced vitamin K–dependent coagulopathy may develop within several days after initiation of therapy. Kodama et al. reported CMZ-induced hypoprothrombinemia developing on day 7 of treatment in a 93-year-old patient,^4)^ whereas Haba et al. reviewed 10 cases and reported that the interval from CMZ initiation to the onset of coagulopathy ranged from 4 to 20 days (mean, 8.7 days).^2)^ In addition, Kimura et al. demonstrated a marked increase in PIVKA-II from 15 to 4697 mAU/mL within 6 days of CMZ administration, supporting the rapid development of vitamin K–dependent coagulopathy following CMZ exposure.^5)^ In the present case, severe coagulopathy developed after approximately 13 days of cumulative CMZ exposure and 6 days after re-exposure on HD 16, which is consistent with the previously reported time course. The occurrence of severe coagulopathy shortly after re-exposure suggests that cumulative CMZ exposure and re-exposure may have contributed to the development of vitamin K–dependent coagulopathy.

Furthermore, a recent multicenter study investigating PT-INR elevation during CMZ therapy identified nutritional risk as an independent predictor of coagulation abnormalities.^6)^ In the present case, severe infection, prolonged fasting, poor nutritional status, and renal dysfunction requiring CHDF may have acted synergistically to increase the risk of CMZ-induced vitamin K–dependent coagulopathy. Therefore, periodic monitoring of PT-INR should be performed during CMZ therapy in high-risk patients, particularly critically ill older patients with malnutrition, prolonged fasting, or renal dysfunction.

A limitation of this case is that mixing studies and individual coagulation factor assays were not performed. Therefore, definitive confirmation of vitamin K–dependent coagulopathy based on coagulation factor analysis was not possible. Nevertheless, the markedly elevated PIVKA-II level, rapid correction of coagulopathy following vitamin K administration, and absence of recurrence after CMZ discontinuation strongly supported the diagnosis.

## CONCLUSIONS

Although CMZ-induced vitamin K–dependent coagulopathy is a rare adverse effect, it can be life-threatening. In high-risk patients, careful monitoring of coagulation parameters, particularly PT-INR, is essential during CMZ therapy. Additionally, CMZ-induced vitamin K–dependent coagulopathy should be considered in the differential diagnosis of unexplained bleeding in critically ill patients.
